# Recombinant human plasma gelsolin reverses increased permeability of the blood–brain barrier induced by the spike protein of the SARS-CoV-2 virus

**DOI:** 10.1186/s12974-022-02642-4

**Published:** 2022-11-24

**Authors:** Łukasz Suprewicz, Kiet A. Tran, Ewelina Piktel, Krzysztof Fiedoruk, Paul A. Janmey, Peter A. Galie, Robert Bucki

**Affiliations:** 1 Department of Medical Microbiology and Nanobiomedical Engineering, Medical University of Białystok, Mickiewicza 2C, 15-222 Białystok, Poland; 2 Department of Biomedical Engineering, Rowan University, Glassboro, NJ 08028 USA; 3 Department of Physiology and Institute for Medicine and Engineering, University of Pennsylvania, Philadelphia, PA 19104 USA

**Keywords:** Plasma gelsolin (pGSN), COVID-19, SARS-CoV-2, Blood–brain barrier, Microfluidics, Tissue engineering

## Abstract

**Background:**

Plasma gelsolin (pGSN) is an important part of the blood actin buffer that prevents negative consequences of possible F-actin deposition in the microcirculation and has various functions during host immune response. Recent reports reveal that severe COVID-19 correlates with reduced levels of pGSN. Therefore, using an in vitro system, we investigated whether pGSN could attenuate increased permeability of the blood–brain barrier (BBB) during its exposure to the portion of the SARS-CoV-2 spike protein containing the receptor binding domain (S1 subunit).

**Materials and methods:**

Two- and three-dimensional models of the human BBB were constructed using the human cerebral microvascular endothelial cell line hCMEC/D3 and exposed to physiologically relevant shear stress to mimic perfusion in the central nervous system (CNS). Trans-endothelial electrical resistance (TEER) as well as immunostaining and Western blotting of tight junction (TJ) proteins assessed barrier integrity in the presence of the SARS-CoV-2 spike protein and pGSN. The IncuCyte Live Imaging system evaluated the motility of the endothelial cells. Magnetic bead-based ELISA was used to determine cytokine secretion. Additionally, quantitative real-time PCR (qRT-PCR) revealed gene expression of proteins from signaling pathways that are associated with the immune response.

**Results:**

pGSN reversed S1-induced BBB permeability in both 2D and 3D BBB models in the presence of shear stress. BBB models exposed to pGSN also exhibited attenuated pro-inflammatory signaling pathways (PI3K, AKT, MAPK, NF-κB), reduced cytokine secretion (IL-6, IL-8, TNF-α), and increased expression of proteins that form intercellular TJ (ZO-1, occludin, claudin-5).

**Conclusion:**

Due to its anti-inflammatory and protective effects on the brain endothelium, pGSN has the potential to be an alternative therapeutic target for patients with severe SARS-CoV-2 infection, especially those suffering neurological complications of COVID-19.

**Supplementary Information:**

The online version contains supplementary material available at 10.1186/s12974-022-02642-4.

## Background

SARS-CoV-2 is a β-coronavirus that mainly causes inflammatory lung pathology associated with thrombosis and increased pulmonary vascular permeability leading to edema and hemorrhage [1]. However, both the cytokine storm during the inflammatory cascade and the spike protein of the virus itself affect other organs, including the brain vasculature [2–5]. Early reports indicate that patients develop a chronic condition characterized by fatigue and neuropsychiatric symptoms, termed long-COVID, now more often called post-acute COVID syndrome (PACS) [6–11]. The most prevalent neurological manifestations of SARS-CoV-2 infection that usually resolve over time in mild cases are headache, anosmia, and dysgeusia [12–15]. However, numerous studies point to other severe complications, such as impaired consciousness, cerebrovascular events, encephalopathy, acute disseminated encephalomyelitis, Guillain–Barré syndrome, strokes, delirium, dementia-like syndrome, and psychiatric disorders, including psychosis, catatonia, and mania [16–18]. Multicenter analyses demonstrated that encephalopathy (up to 42%) and cerebrovascular events (up to 62%) account for most of the COVID-19-associated neurological complications, with inflammatory syndromes, i.e., encephalitis (up to 13%) and Guillain–Barré (up to 9%) considerably less frequent [18, 19]. The occurrence of ischemic stroke is moderately high. It most frequently occurs in younger patients, with more recurring vascular occlusion and higher morbidity than described in patients without COVID-19 and those with influenza. Stroke and inflammatory syndromes seem to have the poorest prognosis for patients of all coronavirus-mediated neurological symptoms [20, 21]. The most common findings detected using neuroimaging enclose leukoencephalopathy, ischemia with large vessel occlusion, encephalitis, hemorrhage in locations not typical for hypertension, and abnormalities in perfusion [22–25]. Microhemorrhages were associated with microvascular disease in post-mortem studies of COVID-19 patients [26, 27]. Those neurological complications accompanying SARS-CoV-2 infection relate directly or indirectly to the preceding impairment of the endothelial barrier, which allows the viral S-protein to pass through the blood–brain barrier and damage of glial cells. Given the range of neurological complications associated with COVID-19, understanding the complex pathogenesis and molecular mechanisms is essential for providing countermeasures for the treatment of SARS-CoV-2 (SARS-2) infections.

The mechanisms by which the coronavirus affects brain vasculature, allowing the virus to cross the blood–brain barrier (BBB) and cause neurological disorders, are not fully understood. Due to the significant genetic similarity between SARS-CoV-2 and other viruses in this group, particularly SARS-CoV-1, specific targets have been identified by which the virus interacts with human cells [28, 29]. So far, it has been demonstrated that the entry and multiplication of the SARS-CoV-2 virus in human cells is initiated by its interaction with receptors located on the cell surface, particularly angiotensin-converting enzyme 2 (ACE2) [30, 31]. Some studies indicate that the interaction of viral proteins with the ACE2 receptor alone is not sufficient to ensure optimal viral entry into cells, and this process may be augmented by other factors/potential targets, such as surface vimentin, heparan sulfate, neuropilins, and sialic acids [32–35]. Recently, RhoA activation has been associated with SARS-CoV-2-mediated barrier breakdown, indicating that the signaling mechanism involves cytoskeletal components [36]. SARS-CoV-2 may also induce the formation of tunneling nanotubes and uses this route to infect cells [37]. The interaction of SARS-CoV-2 Spike protein with Toll-like receptors (TLR-2, TLR-4) is reported to induce a pro-inflammatory response contributing to hyperinflammation [38–40].

Gelsolin is a calcium and phosphatidylinositol 4,5-bisphosphate (PIP_2_)-regulated protein with the ability to cap and sever actin filaments, and low levels of plasma gelsolin, one of the two main isoforms of the protein, are present in patients with severe COVID-19 [41, 42]. Under physiological conditions, the concentration of pGSN in human blood is 150–300 µg/mL, and it is also present in several other body fluids such as lymph, cerebrospinal fluid, and synovial fluid [43–45]. Decreased plasma gelsolin concentration described in critically ill patients and those requiring immediate medical attention, e.g., patients with acute trauma, multi-organ injuries, as well as in the course of brain and liver injury, myocardial infarction and necrosis, septic shock, and SARS-CoV-2 infection correlates with poor clinical outcome [46–49]. The severity of a decrease in pGSN concentration in the blood (hypogelsolinemia) correlates with the course of the disease, higher mortality rates, and the length of stay in intensive care units among patients after major trauma [50]. Recently, the administration of recombinant human plasma gelsolin (pGSN) has been associated with full recovery of an intubated patient with acute COVID-19 pneumonia [51]. Fewer patients with COVID-19 pneumonia required intubation and had severe adverse events (SAEs) when treated with pGSN compared to the control group in a recent blinded, randomized study [52]. Considering the pleiotropic effects of human plasma gelsolin and its contribution to a patient’s recovery from acute Covid-19, pGSN repletion by administration of recombinant protein may also counteract SARS-CoV-2-mediated inflammation, including inflammation within the brain vasculature.

Here, we aimed to evaluate the ability of human recombinant plasma gelsolin to mitigate blood–brain barrier disruption in the presence of the SARS-CoV-2 spike protein S1 subunit (S1). To test the functional outcome of simultaneous addition of S1 and pGSN, we used 2D and 3D in vitro models of the BBB consisting of human cerebral microvascular endothelial cells (hCMEC/D3). We determined the effect of the pGSN on S1-mediated disrupted barrier integrity by measuring the permeability to low molecular weight dextran and transendothelial electrical resistance. In addition to the functional conditions, we evaluated the levels of inflammatory mediators, tight junction proteins, gene expression of signaling pathways, and the motility of endothelial cells. Overall, these studies assess the therapeutic potential of pGSN in protecting the neurological symptoms of COVID-19.

## Materials and methods

### Recombinant human plasma gelsolin

Recombinant human plasma gelsolin (pGSN) used in our study was produced in *E. coli* and provided by BioAegis Therapeutics (North Brunswick, USA).

### Cell culture

Immortalized human cerebral microvascular endothelial cells (#CLU512-A, Cedarlane Laboratories) were used between passage 25 and 35. Cells were cultured in EBM-2 medium (#CC-3156, Lonza) containing 5% FBS (#P30-8500, PAN Biotech), 1.4 µM hydrocortisone (#H0135, Sigma), 10 mM HEPES pH 7.4 (#15630-080, Life Technologies), 5 µg/mL ascorbic acid (#A4544, Sigma), 1% antibiotic antimycotic solution (#A5955, Sigma), 1% chemically defined lipid concentrate (#11905031, Life Technologies), and 1 ng/mL basic fibroblast growth factor (#F0291, Sigma). Cells were grown on collagen type I (0.1 mg/mL, #3443-100-01, R&D Systems)-coated plates at 37 °C with 5% CO_2_, 95% fresh air, and saturated humidity. Cells were seeded at 3 × 10^4^/cm^2^, trypsinized (T4174, Sigma), and replated when approximately 80–90% confluency was reached. The cell culture medium was changed every 2–3 days.

### BBB permeability and TEER testing using a 2D model

**Fig. 1:**
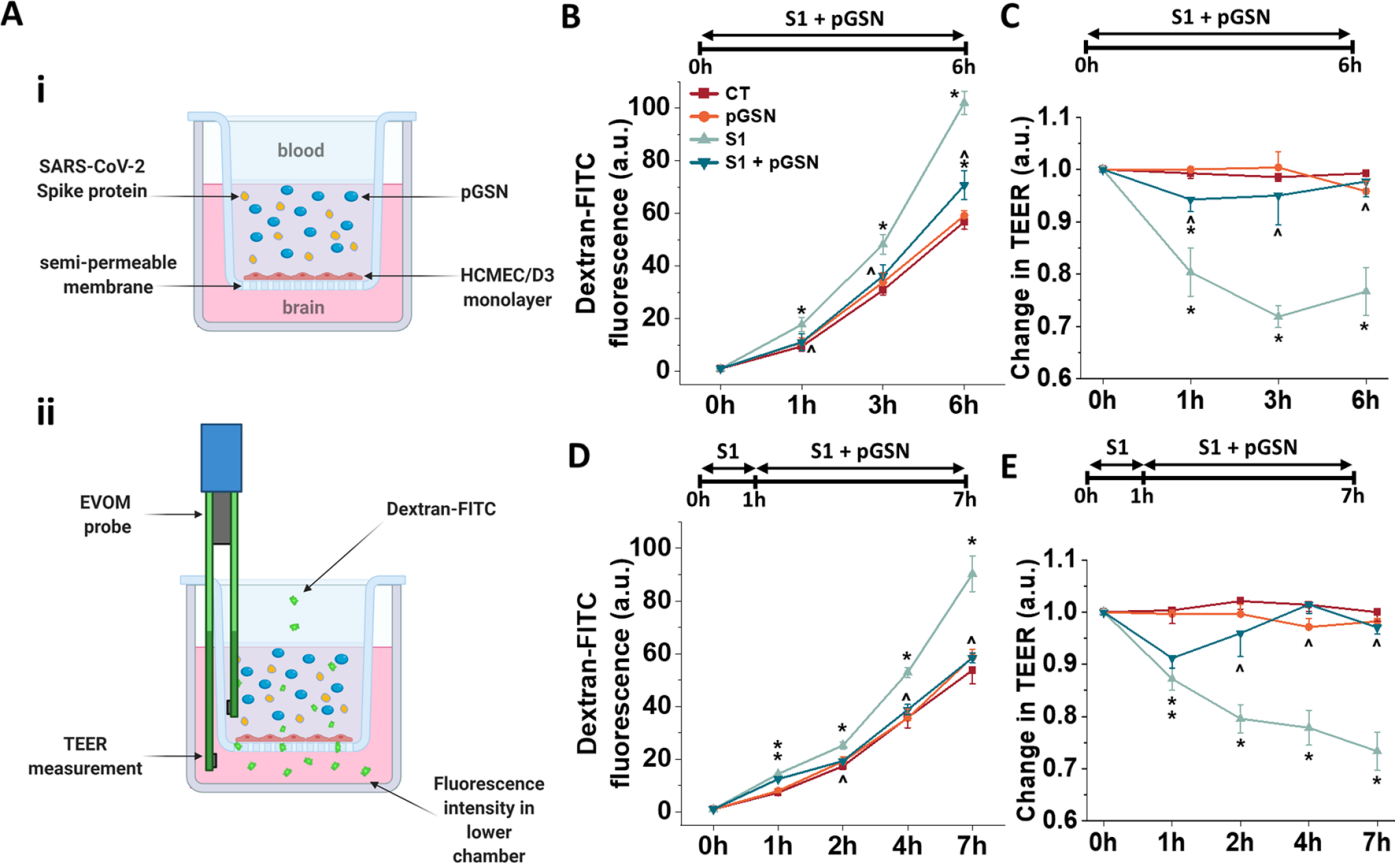
Plasma gelsolin (pGSN) significantly reduces disruption of the blood–brain barrier caused by the SARS-CoV-2 Spike protein S1 subunit in the 2D Transwell permeability assay. Confluent monolayer of human cerebral microvascular endothelial cells (hCMEC/D3) seeded on a Transwell semi-permeable membrane exposed to tested compounds added to the upper chamber (blood) (Panel **A**i). The functional state of the cells as a barrier was evaluated with transendothelial electrical resistance (TEER) measurement and Dextran-FITC permeability assay (Panel **A**ii). The dextran-FITC intensity was measured in the lower chamber (brain) that migrated from the upper chamber (blood) in time (Panels **B**, **D**). Change in TEER was measured using Epithelial Voltohmeter EVOM2 (Panels **C** and **D**). The data represent the mean ± SEM of four (*n* = 4, with 2 inserts used per condition each time) independent experiments. * and ^ indicate statistical significance at *p* ≤ 0.05 compared to CT and S1, respectively, by one-way ANOVA and Tukey post hoc test

To mimic 2D blood–brain barrier functions under static conditions, cells were seeded at a density of 10^4^ cells per transwell insert (#3470, Corning) coated with collagen I (pore size 0.4 μm, diameter 0.33 cm^2^) in 200 μL of complete growth medium. Basolateral chambers were filled with 500 μL of complete growth medium. The medium was changed every 3 days. After the confluent monolayer was formed, cells were washed, and when required, monolayers were incubated with 250 µg/mL pGSN, 10 nM SARS-CoV-2 subunit S1 (#230-01101-100, RayBiotech) and the combination of 250 µg/mL pGSN + 10 nM S1 for 6 h. In all experiments, spike protein S1 subunit was suspended in PBS at 1000 nM concentration and added to cells at a 1 to 100 ratio. To keep the concentration of growth factors at the same level, an equal volume of PBS was added to untreated conditions. The same situation occurred when adding pGSN at a concentration of 10 mg/mL (40x). The schematic representation of the 2D BBB setting is presented in Fig. 1A.

To determine monolayer permeability, a fluorescent molecular tracer assay was performed. To do so, 4-kDa dextran-FITC (#46944, Sigma) was added to the apical chamber to the final concentration of 1 mg/mL, fluorescence in the basolateral chambers was recorded before addition (t_0_) of agents and 0.5, 1, 3, and 6 h later using a Varioskan Lux microplate reader (ThermoFisher). Percent permeability was calculated as the relative fluorescence of medium in the pGSN, S1, and pGSN + S1 treated vs. untreated cells.

Additionally, to evaluate the functional performance of an intact barrier in vitro, the transendothelial electrical resistance (TEER) was measured using an EVOM voltohmmeter (World Precision Instruments) equipped with an STX‐2 chopstick electrode. TEER was measured before adding the agents and 0.5, 1, 3, and 6 h later. Transwell® inserts without cells but coated with rat tail collagen type I, containing EBM-2 medium, were measured and set as blank (approximately 90 Ω). Barrier resistance readings (Ω) were obtained for each well individually and, after subtracting the resistance of the blank, were multiplied by the membrane area (0.33 cm^2^) to calculate Ω*cm^2^. To normalize the data, resistance at *t* = 0 of every single well was set as 1.0. Values treated with pGSN, S1, and pGSN + S1 were normalized to the untreated culture as a control.

### Microfabrication

**Fig. 2:**
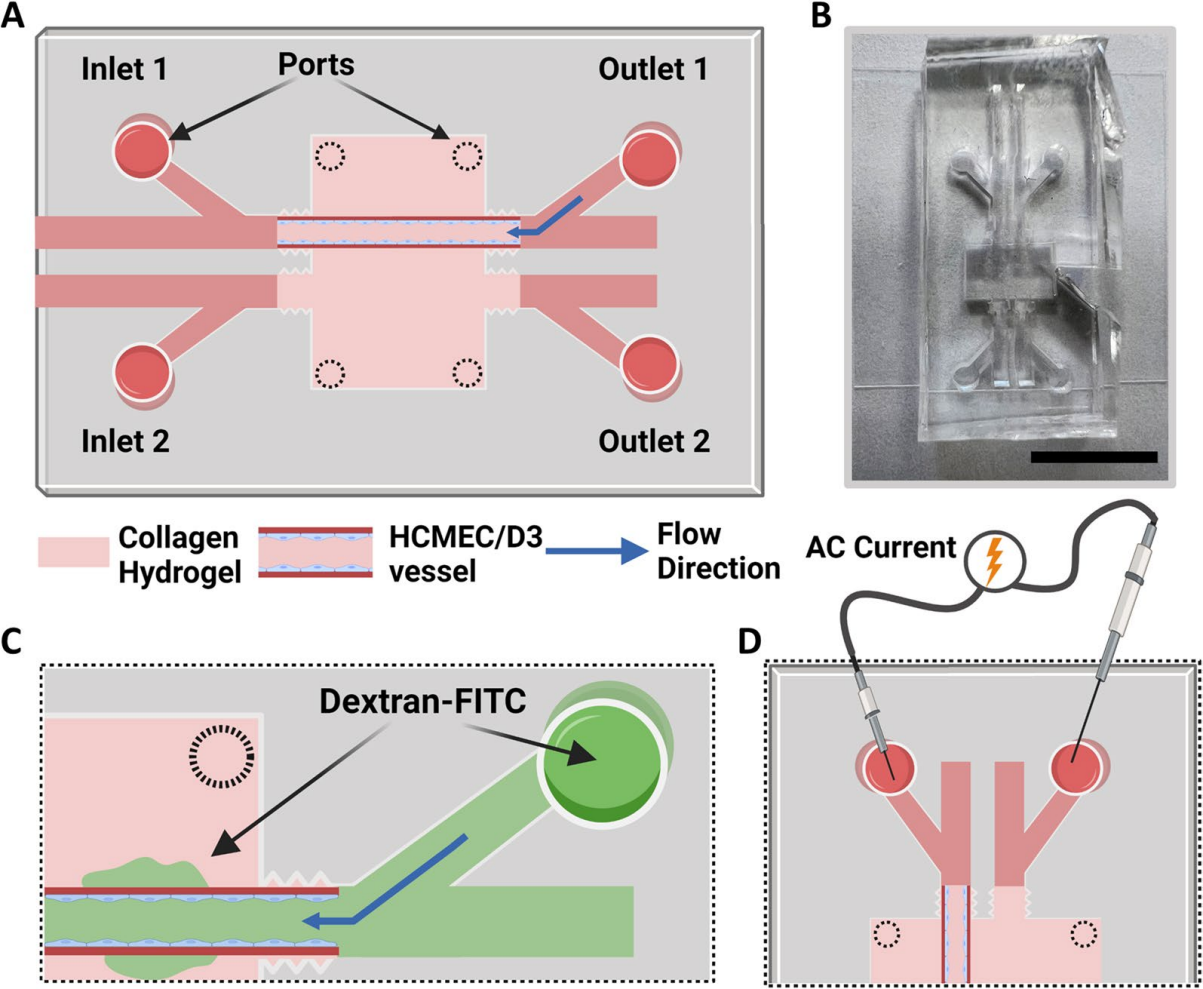
3D blood–brain barrier model. **A** Schematic of a microfluidic device with the location of inlet/outlet ports and ports to fill the hydrogel reservoir. **B** Picture of the PDMS device. Scale bar, 1 cm. **C** Permeability testing with 4 kDa Dextran-FITC configuration. **D** TEER measurements

Fabrication of the microfluidic device was completed using a previously described method [53]. Briefly, SU-8 2025 epoxy-based photoresist (MicroChem) was poured on top of a 3-in. silicon wafer at 95 °C and left overnight. A photomask was applied over the wafer before exposure with a 200-W UV lamp for 2 h. Propylene glycol methyl ether acetate (PGMEA) (MicroChem) was used to dissolve the unpolymerized photoresist, and polydimethylsiloxane (PDMS, # 2065622, Ellsworth) was used to create negative molds from the silicon master. Similarly, positive stamps made of PDMS were cast to create the microfluidic channels on glass coverslips. Before gel fabrication, the hydrogel reservoir was filled with 5 M sulfuric acid (#258105, Sigma) for 90 min, washed several times with distilled water, and then filled with 50 µg/mL collagen type I and sterilized with UV light. The schematic design of the microfluidic device is shown in Fig. 2A, B.

### Blood–brain barrier—a 3D model

Each device was filled with 60 µL hydrogel consisting of 6 µL 10xPBS (#5493, Sigma), 6 µL 0.1 M NaOH (#BA0981118, POCh SA), 18 µL distilled water, and 30 µL 10 mg/mL collagen type I. This formulation yielded a gel consisting of 5 mg/mL collagen. All components were held on ice until mixing, then injected into the hydrogel reservoir of the device. Two 180-mm-diameter acupuncture needles, freshly coated with sterile 0.1% bovine serum albumin (BSA, #A8412, Sigma), were inserted into the needle guides of the device, and the devices were transferred to 37 °C for 10 min to facilitate polymerization. PBS was then pipetted on the ports of the reservoir to prevent the drying of the hydrogel. After 2 h of incubation, the needles were carefully pulled from the hydrogel to create cylindrical voids mimicking the geometry of human vessels (growth area ~ 2 cm^2^). Human cerebral microvascular endothelial cells (hCMEC/D3) were injected into one of the channels at a density of 10 million cells per mL and left for 10 min incubation before an additional 10-min incubation in an inverted position to facilitate full coverage of the cylindrical void. Cell seeded devices were placed in a well of 6-well culture plates containing 7 mL complete growth medium. In the next step, channels were exposed to shear stress by attaching a 20-mL plastic syringe (BD) containing complete EBM-2 secured to a GenieTouch linear syringe pump (Kent Scientific) to the input port for the cell-seeded channel. The syringe pump was set to deliver a 2.4 µL/min volumetric flow rate, which was designed to exert approximately 0.7 dyn/cm^2^ of shear stress on the endothelium within the channel. Vessels were perfused for four days. After 4 days, when hCMEC/D3 monolayer exerts fully developed barrier functions, 20 mL syringes with EBM-2 were substituted with media containing EBM-2 for control conditions, 10 nM S1, and 250 µg/mL pGSN + 10 nM S1 in 5 mL plastic syringes. Flow with these agents was applied in the same conditions for an additional four hours. After 4 h, channels were removed from their testing condition and either assessed for permeability or fixed for immunofluorescence staining.

### Permeability testing in 3D model

Channels were placed on the stage of an inverted epifluorescence microscope (Zeiss Axiostar 10). The devices were perfused with 4-kDa FITC-dextran at a 5 µL/min flow rate. Images were taken at 10-s intervals and then analyzed using ImageJ. Quantification of the permeability coefficient was determined using a method described in a previous study [54]. Briefly, the coefficient was determined by correlating dextran concentration to the fluorescence intensity and inputting the measurements into the following equation:

$$P=\frac{{\mathrm{d}}I}{{\mathrm{d}}t} \frac{r}{{2I}_{0}}$$

(*P*—permeability coefficient, d*I*/d*t*—change in fluorescence intensity in a region of interest outside the lumen, *r*—the channel radius, and *I*_0_—the maximum intensity in the lumen during the duration of the test).

### TEER measurements (3D)

Impedance was measured using a Stingray DS1M12 USB oscilloscope adapter (USB Instruments). The instrument combines the functions of a signal generator and an oscilloscope. Using the DS1M12 EasyScope II software, the output voltage was set to a sine wave with 220 mV amplitude, with electrodes inserted into the input ports of the device so that the measured current passed across the endothelium. The leads of input A measured the voltage across a 460 Ω reference resistor in series with the applied voltage to verify the scope output. The charges of input B were positioned as an ammeter across the reference resistor to indicate the current passing between electrodes. The output voltage frequency was swept from 15 Hz to 15.6 kHz, with intermediate values of 50 Hz, 105 Hz, 558 Hz, and 3.906 Hz, to assure that the impedance followed a characteristic frequency dependence for cell monolayer measurements. The impedance, *Z*, was characterized by calculating the ratio of amplitudes between the applied voltage and measured current and the phase shift between the two waveforms for each frequency. Impedance was defined as: *Z* = *V*/*I* (*V*—the voltage magnitude, *I*—the current magnitude). TEER values were determined as the difference in impedance at 15 Hz, where the capacitance of the electrodes dominates the overall impedance, and at 15.6 kHz, where the resistance of the culture medium is the primary component of the impedance. These values were then subtracted from the impedance difference measured between the inlet and outlet of the device. Measurements in an acellular device were used as a blank and further subtracted from cellularized conditions.

### Confocal microscopy

Devices were fixed in 3.7% paraformaldehyde for 20 min at room temperature. After fixation, the hydrogels were cut from the device hydrogel reservoir and placed in a 100 µL centrifuge tube. Cells within the hydrogel were permeabilized by adding 0.2% Triton X-100 for 30 min at room temperature. The hydrogels were blocked in 3% BSA for 30 min at 37 °C. Hydrogels were then incubated for 48 h at 4 °C with 1:200 anti-ZO-1. After thorough washing, the hydrogels were incubated with 1:1000 of the secondary antibody for one hour and 1 mg/mL DAPI for 15 min at 37 °C in the dark. Images were acquired on an Eclipse Ti-E inverted microscope with an integrated C2þ laser scanning confocal system. Detailed list of used antibodies can be found in Additional file 1: Table S1.

### Migration assay

A scratching assay was implemented to determine the impact of pGSN and S1 on the migration of blood–brain barrier-forming cells. Briefly, hCMEC/D3 cells were cultured in 96-well collagen-coated plates until a confluent monolayer was formed, and homogeneous linear scratches were made using the IncuCyte® WoundMaker (Sartorius AG). PGSN, S1, and pGSN + S1 were added directly to wells, capturing images for 72 h. After that, the images were processed by defining a scratching mask and a cell confluence mask using the IncuCyte® Cell Migration Analysis Software.

### Magnetic bead-based ELISA

Secretion of IL-2, IL-6, IL-8, INF-γ, TNF-α, and GM-CSF was assessed using the Bio-Plex Pro Human Cytokine Assay (Bio-Rad). hCMEC/D3 cells were cultured on 6-well collagen-coated plates until full confluency was reached. PGSN, S1, and pGSN + S1 were then introduced, and the supernatant was collected after 6 and 24 h of incubation. Results were compared to untreated control, normalized to 1.0, and presented as a fold change in cytokine secretion.

### Western blotting

hCMEC/D3 confluent monolayers seeded in 6-well collagen-coated plates were treated in static conditions with pGSN, S1, and pGSN + S1 for 24 h and briefly rinsed with PBS, detached with trypsin, transferred to Eppendorf tubes, and centrifuged. The supernatant was discarded, and the whole-cell lysate was prepared using RIPA lysis buffer (#89901, ThermoFisher) with Pierce Protease Inhibitor (#A32963, ThermoFisher) added freshly before use. Cells were lysed for 20 min on ice and centrifuged at 14,000 rpm for 20 min at 4 °C. Next, supernatants were transferred to fresh tubes and the Bradford (#5000006, Bio-Rad) assay was performed to determine protein concentration. Lysates were subjected to electrophoresis using 10% sodium dodecyl sulfate–polyacrylamide (SDS-PAGE) at a 15 µg per lane concentration. After SDS-PAGE separation, proteins were blotted onto polyvinylidene fluoride membranes. Next, the membranes were submerged in methanol, then blocked for 1 h in 5% nonfat dry milk in TBS-T (150 mM NaCl, 50 mM Tris base, 0.05% Tween 20, pH = 7.4). Blocked protein blots were incubated with rabbit anti-occludin (1:200), mouse anti-claudin 5 (1:500), mouse anti-VE cadherin (1:500), rabbit anti-ZO-1 (1:200), mouse anti-β-catenin (1:300), rabbit anti-VEGFR2 (1:500), and mouse anti-β-actin (1:5000), in TBS-T at 4 °C overnight, followed by incubation with goat anti-rabbit IRDye 800CW IgG and goat anti-mouse IRDye 800CW IgG secondary antibody in TBS-T (1:20,000) at room temperature for 1 h, in the dark. Protein blots were visualized with the Odyssey LiCor Imaging System (LiCor Biosciences). Band intensities were quantified using Image Studio Acquisition Software. Data are presented as relative intensity of protein of interest bands in pGSN, S1, or pGSN + S1-treated samples compared to the untreated samples and normalized to β-actin. Detailed list of used antibodies can be found in Additional file 1: Table S1.

### qRT-PCR

Confluent cell monolayers were treated with pGSN, S1, and pGSN + S1 for 6 h and briefly rinsed with PBS. Total RNA was extracted using the Universal RNA Purification Kit (#E3598-02, EurX). The concentration and purity of isolated RNA were evaluated using a Qubit 4 fluorometer (ThermoFisher). cDNA was synthesized with 100 ng of RNA in a 20-μL reaction mix using the iScript™ cDNA Synthesis Kit (#1708891, Bio-Rad). qRT-PCR was performed with 20 ng of cDNA in a 20-μL reaction containing SsoAdvanced Universal SYBR® Green Supermix (#1725274, Bio-rad) using VEGF signaling and activation PrimePCR plates (#10025756, Bio-Rad) on CFX Connect Real-Time PCR Detection System (Bio-Rad). GAPDH was used as an internal control. Gene expression levels were reported as relative quantity, expressed as 2^−ΔΔCt^, and are presented as Log_2_FC.

### Statistical analysis

Quantitative data are expressed as mean ± SEM. Statistical analyses were evaluated using the one-way ANOVA with Tukey’s post hoc test, with *p* ≤ 0.05 considered statistically significant.

## Results

### pGSN inhibits SARS-CoV-2 S1-induced increase in permeability of BBB in the 2D model

The main feature of the blood–brain barrier is the presence of endothelial tight junctions that produce highly selective permeability of the barrier. The S1 subunit of the SARS-CoV-2 spike causes disruption of these tight junctions and a subsequent barrier breakdown. We performed permeability assays to determine whether pGSN could mitigate tight junction disassembly in the presence of the S1 subunit. 4-kDa FITC-dextran was used to focus on paracellular passage via cellular junctions and exclude other forms of active transport, e.g., transcytosis (Fig. 1A). Data presented in Fig. 1B and D show the mean change of fluorescence in arbitrary units (a.u.) ± SEM. To better visualize changes in permeability, the fluorescence of untreated hCMEC/D3 cells at each time point (1, 3, and 6 h) was normalized to 100%. We did not observe a statistically significant change in permeability of the BBB upon the addition of pGSN (250 µg/mL), which is approximately 20 times more than the pGSN concentration in a cell growth medium with 5% serum. At 1 h, 10 nM SARS-CoV-2 S1 increased permeability by 188%. When 250 µg/mL pGSN was added at the same time as 10 nM S1 subunit, the permeability change dropped to 117%, which is statistically indistinguishable from the control value. After 3 h, 10 nM SARS-CoV-2 S1 gives a rise to 157%, while 250 µg/mL pGSN + 10 nM of S1 limited this effect to 118%, a 39% decrease in permeability compared to spike protein S1 subunit alone. At 6 h, 10 nM S1 induced 180% permeability, 250 µg/mL pGSN + 10 nM of S1 induced 124%, a 55% less than S1. To test whether gelsolin could reverse a permeability change already initiated by S1, endothelial cells were preincubated for 1 h with protein S1 before pGSN addition. As shown in Fig. 1D, the addition of pGSN stopped further deterioration of the BBB caused by spike protein and returned permeability levels to those of the control after 1, 3, and 6 h. Thrombin, an inducer of endothelial permeability, was used as a positive control to evaluate experimental settings, while human albumin was used as a negative control (Additional file 1: Fig. S1). Thrombin, as expected, caused a significant increase in blood–brain barrier vascular permeability comparable to S1 during the initial phase of stimulation (up to 3 h). However, after 6 h, vascular permeability was lower than with S1, probably because of the short half-life of thrombin. Human albumin did not inhibit thrombin or S1 protein, nor did it alter endothelial permeability in the condition without additional stimulation.

To provide further evidence of the protective effects of pGSN on the BBB, transendothelial electrical resistance (TEER) measurements were performed. TEER values assess the functional status of brain endothelium; higher resistance denotes decreased permeability, while lower resistance indicates compromised permeability. Baseline TEER values of approx. 30 Ω*cm^2^ were required prior to the onset of testing, and all measurements were normalized to this initial measurement (TEER at 0 h = 1.0). As shown in the untreated control conditions (Fig. 1C), TEER values were constant throughout the entire experiment. Similarly, 250 µg/mL pGSN samples did not vary significantly from the control. 10 nM SARS-CoV-2 S1 significantly reduced the resistance at all time points, while the addition of 250 µg/mL pGSN mitigated the reduction in TEER measurements. When added 1 h after S1, pGSN not only stopped the decreased resistance caused by S1, but returned TEER values to their control levels within 3 h (Fig. 1E).

The permeability and TEER measurements suggest that pGSN exerts a protective effect on blood–brain barrier integrity by reversing the increased permeability caused by SARS-CoV-2 S1 in static conditions. The use of small, positively charged molecular tracers points to the broken intercellular junctional proteins as a port of S1 protein entry for the central nervous system [55]. Since pGSN was found to promote TJ formation within brain vasculature, this pGSN ability might be delaying or repairing barrier disruption.

### pGSN prevents blood–brain barrier disruption caused by SARS-CoV-2 S1 protein in the 3D model

**Fig. 3:**
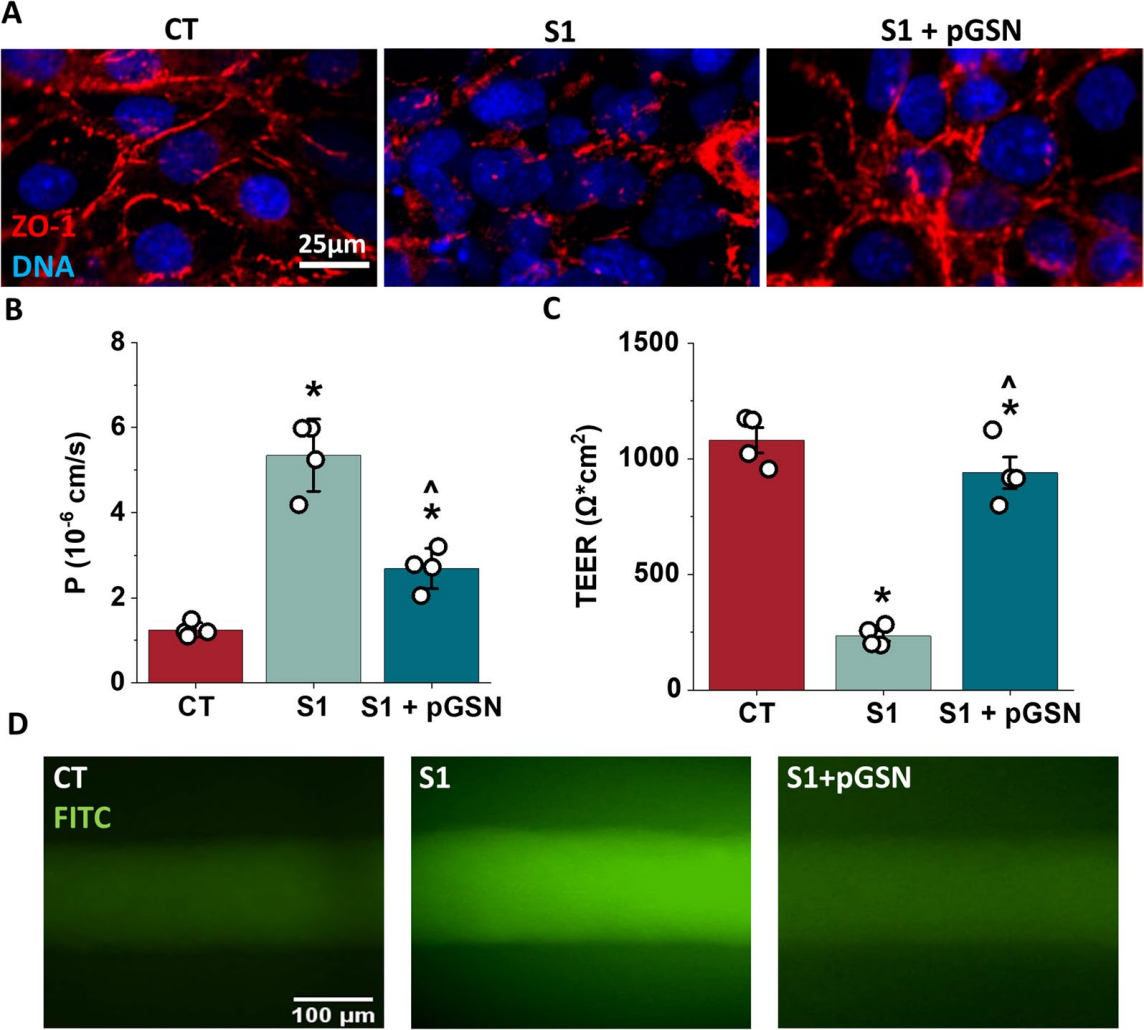
Plasma gelsolin reverses the destructive effect of SARS-CoV-2 Spike protein S1 subunit on blood–brain barrier function in the 3D flow model. Confocal images of hCMEC/D3 cells (panel **A**), tight junction protein ZO-1 (red), and nuclei (blue). Permeability coefficient measured from dextran experiments for endothelial channels exposed to S1 and S1 + pGSN (Panel **B**). TEER measurement results (Panel **C**). Images demonstrating the measurement of vessel permeability using 4 kDa FITC-dextran (green) and the effects of the S1 and S1 + pGSN (Panel **D**). Barrier permeability and TEER measurement were performed after 4 h of perfusion with 10 nM of S1 and S1 + pGSN. The data represent the mean ± SEM of four independent experiments (*n* = 4). * and ^ indicate statistical significance at *p* ≤ 0.05 compared to CT and S1, respectively, by one-way ANOVA and Tukey post hoc test

A three-dimensional microfluidic model of BBB was used to determine to verify that pGSN protects the barrier in the presence of the S1 subunit in a microenvironment that better mimics in vivo vasculature (Fig. 2A, B). Moreover, the 3D model can also incorporate crucial components of in vivo vasculature, including the application of shear stress. The 3D BBB model was perfused for 4 days with 0.7 dyn/cm^2^. Subsequently, vessels were perfused for four hours in the same fluid shear stress conditions (0.7 dyn/cm^2^) with cell culture medium, 10 nM SARS-CoV-2 S1, or 10 nM SARS-CoV-2 S1 + 250 µg/mL pGSN. Permeability of the BBB in the 3D model was performed using the molecular tracer assay and TEER measurements (Fig. 2C, D). Additionally, after 4 h perfusions, vessels were immunostained to examine zonula occludens-1 (ZO-1), an indicator of barrier breakdown, acting as a scaffold in the tight junction complex. In Fig. 3A, ZO-1 forms a sharp pattern localized at the cell–cell contact area in untreated conditions. Perfusion with 10 nM of SARS-CoV-2 S1 protein disrupts ZO-1 localization, potentially contributing to impaired barrier permeability. Simultaneous perfusion with 10 nM SARS-CoV-2 with 250 µg/mL pGSN protects ZO-1 protein from disruption, with a pattern resembling a branching network of sealing strands, similar to untreated control. Assessment of barrier permeability with 4 kDa Dextran-FITC (Fig. 3B) indicated over a threefold increase in the permeability coefficient following exposure to 10 nM SARS-CoV-2 S1, while the addition of 250 µg/mL pGSN reduced by nearly twofold leakage of BBB caused by S1. Images of the vessels following dextran perfusion validated the permeability measurements; untreated vessels exhibited a sharp fluorescence gradient at the vessel wall, and S1-treated vessels showed substantial drainage, inhibited during simultaneous addition of pGSN (Fig. 3D). TEER measurements show a fivefold decrease in electrical resistance after perfusion with 10 nM of SARS-CoV-2, while TEER with S1 + pGSN was higher by fourfold than with S1 subunit alone. Overall, findings in the 3D model support the results of the 2D studies demonstrating a protective effect of pGSN during SARS-CoV-2 S1 subunit-mediated barrier breakdown.

### Inhibition of hCMEC/D3 cell migration caused by S1 protein is reversed by pGSN

**Fig. 4:**
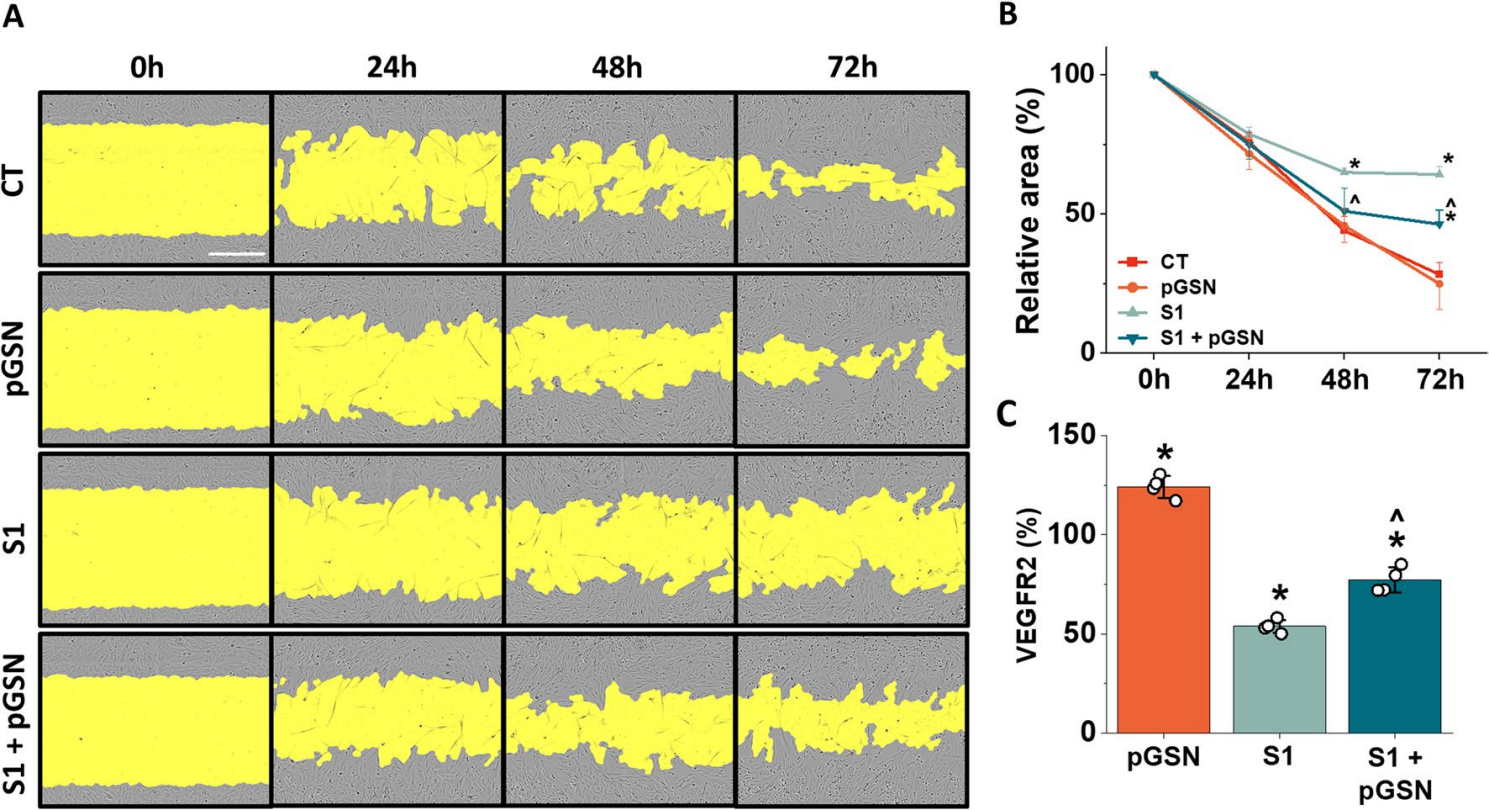
Plasma gelsolin improves migration of hCMEC/D3 upon SARS-CoV-2 Spike protein S1 subunit treatment in wound-healing assay. Images of endothelial cells in a wound healing setting (Panel **A**), the yellow color indicates wound width, which is quantitatively shown in Panel **B**. Western blot quantitative analysis of VEGRF2 expression in hCMEC/D3 cells after 24 h stimulation with pGSN, S1, and S1 + pGSN (Panel **C**). The data represent the mean ± SEM of four independent experiments (*n* = 4). * and ^ indicate statistical significance at *p* ≤ 0.05 compared to CT (100%) and S1, respectively, by one-way ANOVA and Tukey post hoc test

The migration of endothelial cells is a crucial process during angiogenesis and vasculogenesis as well as in a damaged vasculature to restore vessel integrity. A wound-healing assay was performed to determine whether SARS-CoV-2 S1 restricts migratory properties within brain vasculature and whether pGSN alters the effect. Wound scratch closure was monitored for 72 h (Fig. 4A). Due to the variable size of the initial scratch, the wound area at time 0 was normalized to 100%, and data were presented as a change from the relative initial wound area. As shown in Fig. 4B, pGSN at 250 µg/mL alone does not affect the migration of the hCMEC/D3 cells during the entire experiment. At 24 h, neither 10 nM SARS-CoV-2 S1 protein nor pGSN significantly changed cell migration. After 48 h, 10 nM SARS-CoV-2 S1 wound size was 21% greater than in untreated conditions, while the addition of pGSN at 250 µg/mL reduced wound area by 14% compared to S1 alone. At 72 h, with 10 nM SARS-CoV-2 S1 protein, wound size was 36% bigger than CT, while wound area after addition of 250 µg/mL pGSN was smaller by 18% than with SARS-CoV-2 S1 alone.

To determine the possible mechanism of preventive pGSN effect when simultaneously added with SARS-CoV-2 S1 protein, lysates of 24 h treated hCMEC/D3 cells were evaluated for VEGFR2 expression using the Western Blot technique. VEGFR2 is a mediator of intracellular signaling involved in cell survival, proliferation, migration, cytoskeleton rearrangement, and vascular permeability. As shown in Fig. 4C with 250 µg/mL pGSN, VEGFR2 expression was recorded at 124%, and with 10 nM SARS-CoV-2 S1 at 50%. The addition of pGSN to S1 restored VEGFR2 expression to 76%, which was 26% higher than S1 alone. We cannot exclude the possibility of direct stimulation of VEGFR2 by pGSN, which may enhance brain endothelial cell migration. However, it seems more likely that the known anti-inflammatory effect of pGSN may indirectly influence the S1-mediated inhibition of hCMEC/D3 migration, contributing to the formation of the tight junction proteins that limit increased monolayer permeability.

### pGSN reduces pro-inflammatory cytokine secretion caused by SARS-CoV-2 S1 protein in the initial phase of cells stimulation

**Fig. 5:**
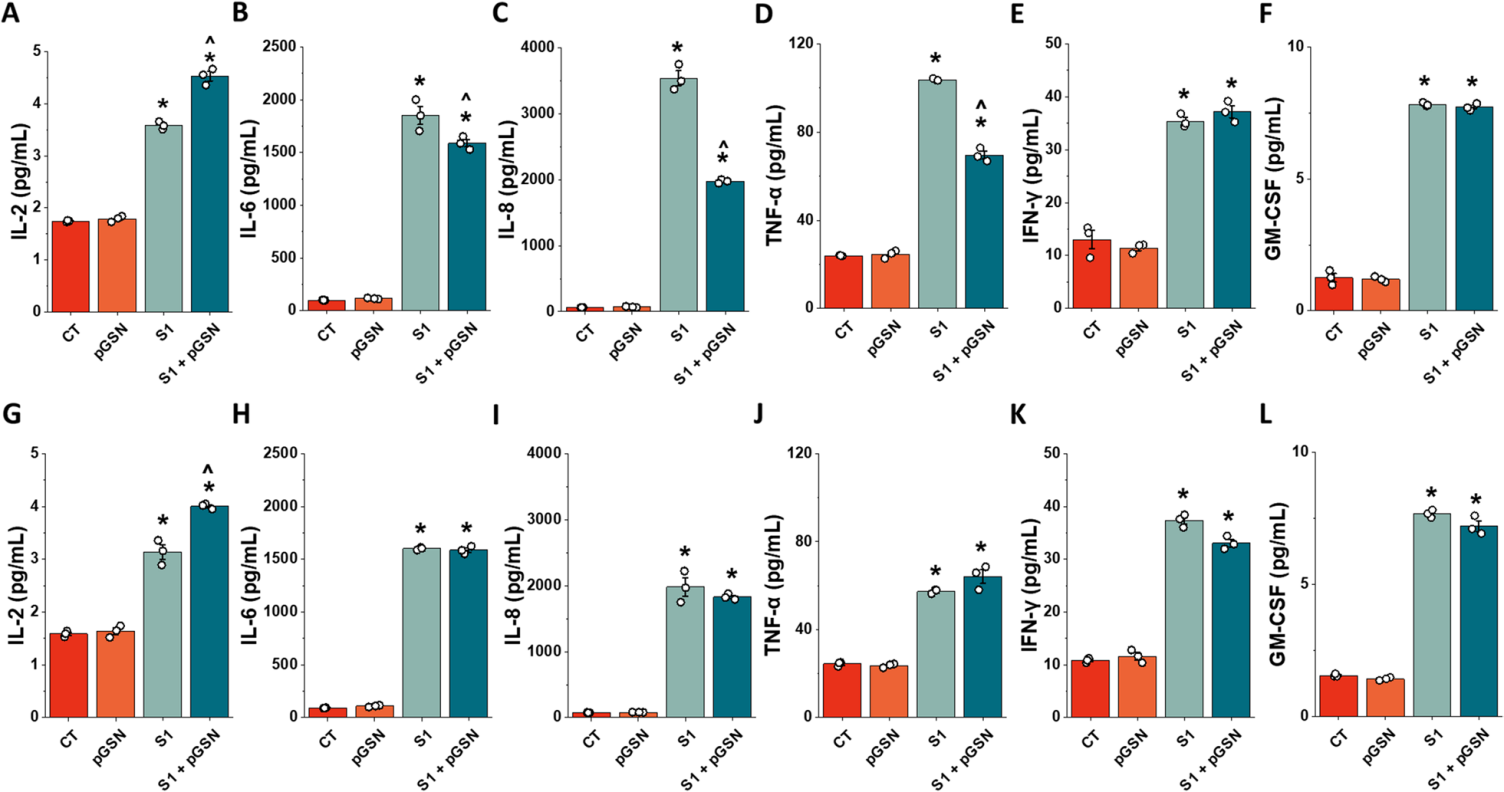
Secretion of inflammatory mediators by hCMEC/D3 cell line stimulated with SARS-CoV-2 Spike protein S1 subunit and S1 + pGSN after 6 and 24 h. Expression of IL-2 (**A**, **G**), IL-6 (**B**, **H**), IL-8 (**D**, **I**), TNF-α (**D**, **J**), INF-γ (**E**, **K**), and GM-CSF (**F**, **L**). Protein expression after 6 h of treatment is presented on Panels **A**–**F**, and expression after 24 h is on Panels **G**–**L**. Alternation of inflammatory response was monitored using a magnetic bead-based assay. The data represent the mean ± SEM of three independent experiments (*n* = 3). * and ^indicate statistical significance at *p* ≤ 0.05 compared to CT and S1, respectively, by one-way ANOVA and Tukey post hoc test

Endothelial dysfunction during COVID-19 is in part caused by a spike protein-induced cytokine storm that involves a cascade release of pro-inflammatory mediators. We performed a magnetic bead-based assay to assess whether plasma gelsolin can reduce S1-induced cytokine secretion from the endothelial cells. Figure 5 represents IL-2, IL-6, IL-8, INF-γ, TNF-α, and GM-CSF secretion upon pGSN, S1, and S1 + pGSN stimulation at 6 and 24 h. The addition of 250 µg/mL pGSN to the hCMEC/D3 confluent monolayer did not lead to significant changes in cytokine secretion after 6 and 24 h. The reductions with pGSN treatment compared to S1 alone are modest after 6 h for most cytokines except for IL-8 and TNF-α as shown in Fig. 5. We did not observe a relevant change in S1-induced secretion of IFN-γ and GM-CSF, while only a slight decrease was noted for IL-6 upon addition of pGSN. At 6 h (Fig. 5A–F), simultaneous addition of 10 nM SARS-CoV-2 S1 and 250 µg/mL pGSN increased by 0.53 fold IL-2 secretion compared to S1 alone. Furthermore, a decrease of 10 nM SARS-CoV-2 S1-induced secretion was noted in IL-6 by 2.59 fold, IL-8 by nearly 25 fold, and TNF-α by 1.42 fold. After 24 h of stimulation (Fig. 5G–L), the addition of gelsolin did not significantly alter cytokine secretion. However, the decrease by 1.96 fold change for IL-8 is worth noticing. Our cytokine secretion assessment data indicate a protective, anti-inflammatory role of plasma gelsolin against SARS-CoV-2 S1-exposed brain endothelium. Plasma gelsolin is particularly effective towards S1-induced IL-8 and TNF-α secretion, both cytokines with a potent ability to impair endothelial permeability that has been widely associated with poor clinical outcomes for patients with COVID-19 [56, 57]. The results are consistent with previous findings suggesting a role for pGSN in regulating cerebral vascular permeability [58].

### Downregulation of tight junction-forming proteins caused by SARS-CoV-2 S1 protein is inhibited by pGSN

**Fig. 6:**
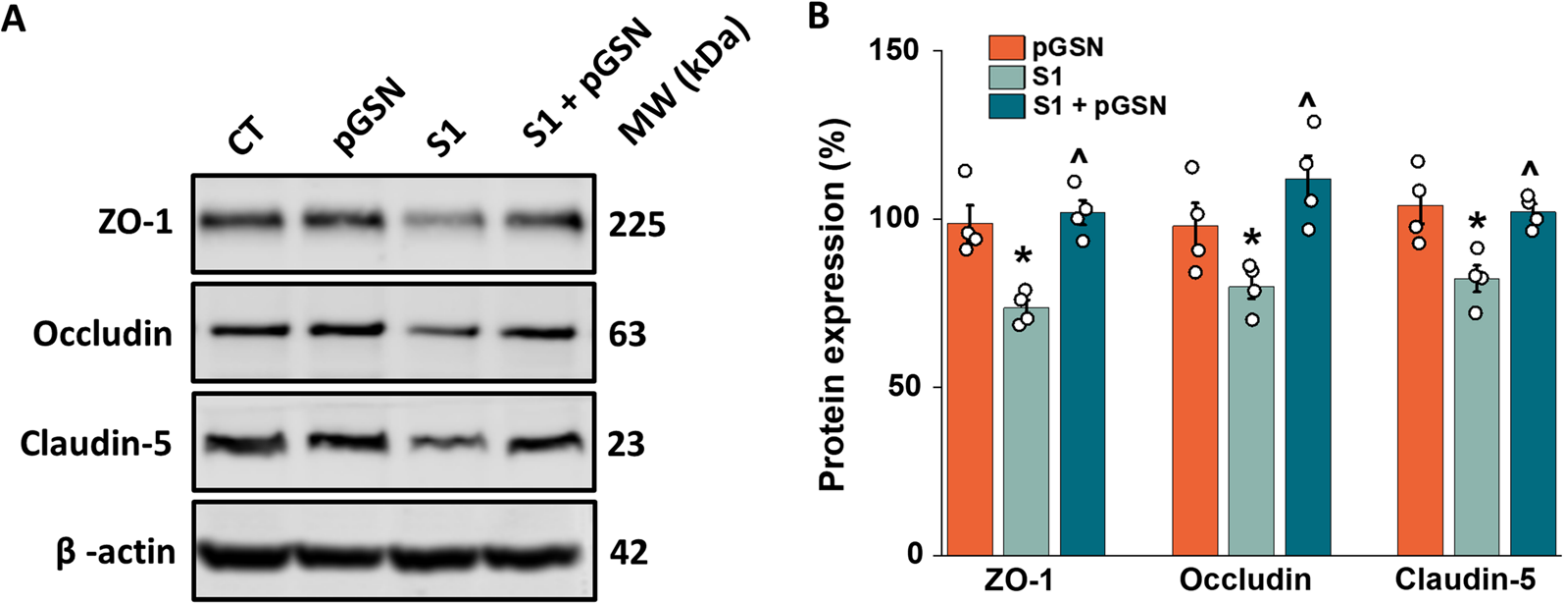
Spike protein S1 subunit selectively decreases cell junction proteins in endothelial cells, forming a blood–brain barrier. Western blot bands (Panel **A**) and protein expression fold change (Panel **B**). The data represent the mean ± SEM of four independent experiments (*n* = 4). * and ^indicate statistical significance at *p* ≤ 0.05 compared to CT (100%) and S1, respectively, by one-way ANOVA and Tukey post hoc test

The main role of the blood–brain barrier is to selectively impede the passage of molecules into the central nervous system through intercellular bonds mainly formed by tight junctions (TJs) and adherens junctions (AJs). These restrictions on the transport across the blood–brain barrier protect the CNS from harmful substances and maintain brain homeostasis. The main TJ forming proteins ZO-1, occludin, and claudins are among the most confining sealing elements formed between brain endothelial cells throughout the organism. In SARS-CoV-2 infection, the endothelium is disrupted by the degradation of junctional proteins that maintain vascular integrity. To test whether pGSN might reverse that trend, TJ and AJ protein expression was evaluated in hCMEC/D3 monolayer upon exposure to 10 nM SARS-CoV-2 S1 for 24 h (Fig. 6). Our findings support previous studies showing the effect of spike proteins on the downregulation of TJs protein expression (Fig. 6A, B) [2, 55]. However, we did not observe alterations in the expression of the AJ forming proteins VE-Cadherin and β-catenin (data not shown), which points to a certain selectivity of spike protein S1 subunit towards proteins involved in the formation of tight junctions. 10 nM SARS-CoV-2 S1 subunit caused a decrease in expression of ZO-1 by 25%, occludin by 20%, and claudin 5 by 16%. In the case of SARS-Cov-2 S1 protein at 10 nM with concurrent addition of 250 µg/mL pGSN, TJ protein expression did not differ compared to the untreated control, an increase in expression relative to S1 for ZO-1 by 26%, occludin by 31% and claudin 5 by 17% was observed. The results demonstrate that the proteins forming tight junctions may serve as molecular targets for SARS-CoV-2 S1 in brain vasculature, and plasma gelsolin may inhibit these proteins' turnover. Moreover, the action of S1 towards TJs appears to be specific due to the lack of downregulation of AJ expression.

### pGSN inhibits activation of NF-κB triggered by S1-protein in endothelial cells

Gene expression studies were conducted to understand the possible mechanism or signaling pathways targeted by the SARS-CoV-2 S1 subunit that pGSN might target. 10 nM SARS-CoV-2 S1 protein and its combination with 250 µg/mL pGSN were added to hCMEC/D3 cells for 6 h in quadruplicates. All samples passed QC standards for gene expression and hybridization. Genes that had at least 125% (± 25%) change in expression and statistical significance at *p* ≤ 0.05 were selected. Changes in gene expression are presented as Log_2_ of Fold Change (Log_2_FC). Our data strongly support previous reports pointing to the PI3K/AKT/MAPK/NF-κB-dependent activation of endothelial cells caused by the S1 subunit of SARS-CoV-2 [40, 59, 60].

**Fig. 7:**
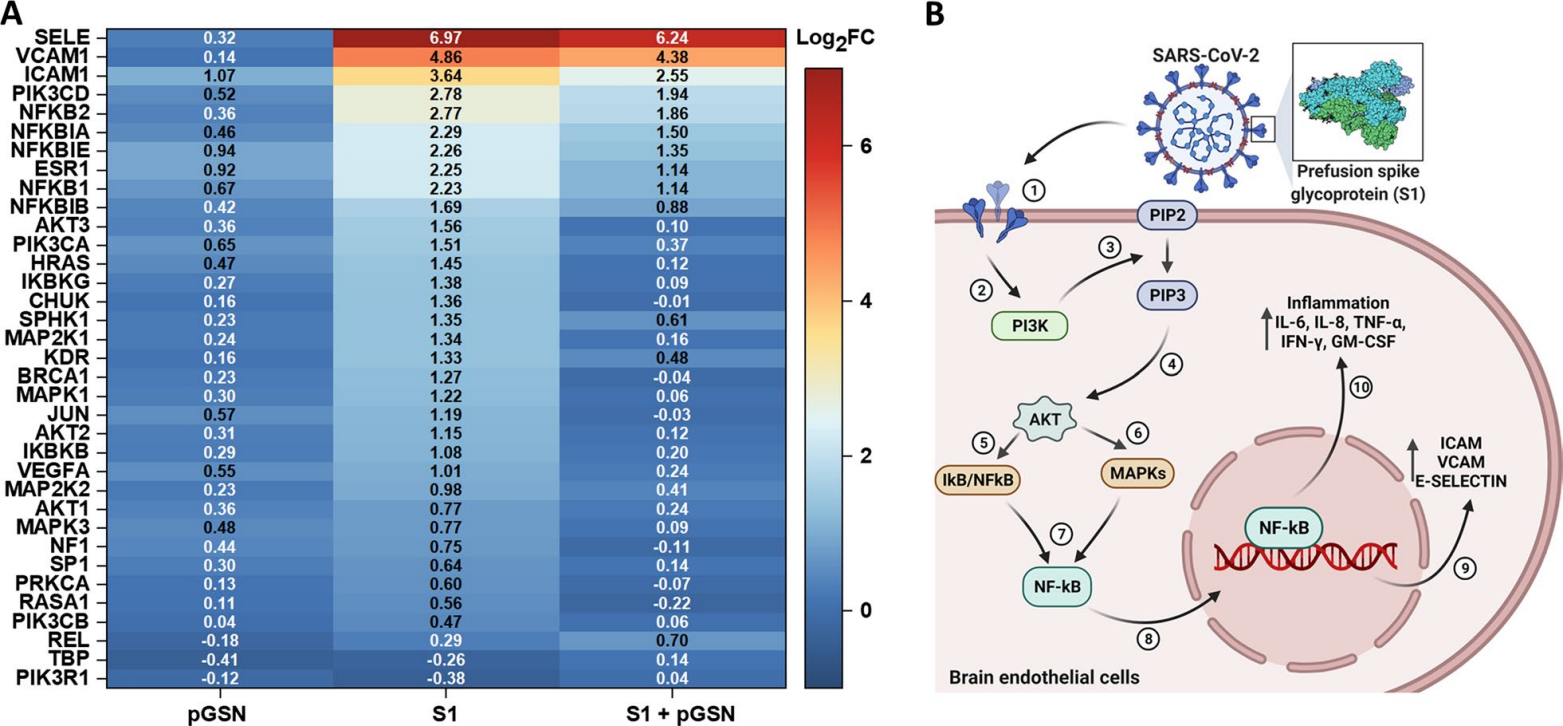
Plasma gelsolin (pGSN) inhibits NF-κB activation by the SARS-CoV-2 Spike protein S1 subunit in hCMEC/D3 cells. Panel **A** shows a Log_2_ fold change heat map for genes involved in VEGF signaling and activation of blood–brain barrier endothelial cells upon 6 h stimulation with pGSN [250 µg/mL], S1 [10 nM] and S1 [10 nM] + pGSN [250 µg/mL]. Log_2_ fold change was calculated based on delta Ct values compared to the control samples. Warmer colors imply increased expression, while cold reflects decreased expression. The assay was performed twice in quadruplicate; data within frames shows Log2FC for every tested gene. Panel **B** shows the schematic representation of signaling pathways triggered by the SARS-CoV-2 Spike protein S1 subunit in hCMEC/D3 cells. (1) Spike protein interacts with the given receptor on a cell membrane, which (2) activates the catalytic effect of PI3K on PIP2, (3) enzymatically transforming it to PIP3, which is possibly inhibited by plasma gelsolin given its direct binding to PIP2. (4) PIP3 binds to AKT, promoting its phosphorylation and activation. (5, 6) Activated AKT regulates transcriptional activity of MAPK kinases and NF-κB by inducing phosphorylation and degradation of inhibitor of κB (IκB). (7) MAPK initiates the downstream induction of NF-κB and its translocation (8) to the nucleus. (9, 10) NF-κB, after activation, triggers the transcription of various genes and thereby regulates inflammation

As shown in Fig. 7A, the simultaneous addition of 250 µg/mL pGSN and 10 nM S1 protein reduced expression of genes involved in PI3K signaling, namely phosphatidylinositol-4,5-bisphosphate 3-kinase catalytic subunit Alpha, Beta and Delta (PIK3CA, PIK3CB, PIK3CD) by 1.13, 0.41 and 0.84 Log_2_FC, respectively.

Significant downregulation of gene expression was recorded for the AKT family when pGSN was administered with S1. RAC alpha serine/threonine-protein kinase 1, 2 and 3 (AKT1, AKT2, AKT3) were downregulated by 0.53, 1.04 and 1.46 Log_2_FC, respectively. Noteworthy, gene expression of all AKT family genes after pGSN treatment did not differ significantly from basal gene expression in an untreated condition.

Inhibition of genes involved in MAPK signaling was also seen during the addition of pGSN. Mitogen-activated protein kinase 1 and 3 (MAPK1, MAPK3), as well as mitogen-activated protein kinase 1 and 2 (MAP2K1 and MAP2K2), were downregulated by 1.16, 0.68, 1.18 and 0.56 Log_2_FC, respectively. From MAPK genes, only MAP2K2 after addition of pGSN differed significantly from gene expression in untreated conditions.

For genes of the NF-κB pathway, we noted a reduction in the expression of inhibitor of nuclear factor kappa-B kinase subunit alpha, beta, and gamma (CHUK, IKBKB, and IKBKG) to the level of untreated control by 1.36, 0.88 and 1.39 Log_2_FC, respectively. Moreover, we noted downregulation in mRNA expression of nuclear factor Kappa B Subunit 1 and 2 (NFKB1, NFKB2) by 1.09 and 0.91 Log_2_FC, respectively. Nuclear factor-kappa-B-inhibitors alpha, beta, and epsilon (NFKBIA, NFKBIB, and NFKBIE) were downregulated by 0.79, 0.81 and 0.91 Log_2_FC, respectively.

As a result of the pro-inflammatory response triggered by NF-κB activation, we encountered upregulation of intercellular adhesion molecule 1 (ICAM1), vascular adhesion molecule 1 (VCAM1), and E-selectin (SELE), that was suppressed by pGSN by 1.09, 0.48 and 0.58 Log_2_FC, respectively.

The cascade of PI3K/AKT/MAPK/NF-κB signaling pathways is schematically presented in Fig. 7B. AKT resides in an inactive conformation in the cytosol until interaction with phosphatidylinositol (3,4,5)-trisphosphate (PIP_3_) activates and translocates it to the plasma membrane [61]. PI3K is necessary for AKT activation by catalyzing the phosphorylation of the endogenous phosphatidylinositol 4,5-bisphosphate (PIP_2_) into PIP_3_ [62]_._ Plasma gelsolin binds to PIP_2,_ which potentially inhibits activation of the downstream AKT-dependent MAPK and NF-κB pathways [44].

Our results strongly suggest a protective role of plasma gelsolin towards the vascular endothelium through inhibition of gene expression of signaling pathways involved in the induction of inflammation, resulting in damage to the brain endothelium and loss of its function as a vascular barrier in SARS-CoV-2 infection.

## Discussion

Identifying the role of pGSN in the pathogenesis of SARS-CoV-2 infection is motivated by reports that pGSN depletion correlates with the severity of COVID-19 [41, 42]. Here, we have shown that pGSN protects brain endothelial cells from loss of barrier function caused by the SARS-CoV-2 Spike protein. Our study has two main limitations. First, the use of the spike protein by itself does not capture any potential effects that plasma gelsolin may have in the complex interaction between the virus and the cell surface. The second limitation is the 2D and 3D models of the BBB that we explore to study BBB functions. With its benefits in dissecting the function of endothelial cells, we have to be aware that the physiological BBB is more complex, and the endothelial cells are accompanied by glial cells and pericytes, which are also essential for the BBB functionality.

Our observations agree with several previous reports indicating a direct effect of SARS-CoV-2 or its components on the permeability of the endothelial cell monolayer [2, 3, 5, 63, 64]. As would be expected, increased release of pro-inflammatory cytokines and disrupted TJ are the main causes of S1 protein-induced BBB leakage [2, 5, 65]. Recently, one study proposed a protective effect of pGSN on the expression of the tight junction protein (ZO-1) in the epithelial cells of the choroidal plexus [66]. These results also bolster our findings by indicating that pGSN can modulate tight junctions during inflammation. Moreover, our study is the first work to date to investigate the anti-inflammatory effects of human pGSN using a 3D microfluidic model of BBB, revealing that TJ structure and function negatively affected by the spike protein may regain their physiological function upon pGSN addition. There is no available data regarding possible direct interaction between pGSN and S1 protein. However, our preliminary dot-blot analysis excluded such interaction (data not shown), suggesting that the protective effect on endothelial cells depends on the immunomodulatory properties of pGSN. Our results indicate that pGSN exerts pleiotropic effects on the BBB and is associated with several different cell signaling pathways. The complex action of pGSN and interaction with molecular mechanisms includes the wound-healing process associated with the ability of pGSN to accelerate cell migration [67, 68], as well as anti-inflammatory and antioxidant properties that may limit the exaggerated inflammatory response of endothelial cells forming the blood–brain barrier. Since the ability of the S1 protein to inhibit cell migration was also reversed by pGSN, we suggest that this property of pGSN was at least partly due to its ability to induce VEGFR2 expression. However, there is a report indicating an increased expression of VEGFA, a factor that promotes endothelial cell migration, in acute lung injury caused by SARS-CoV-2 infection [69], which is also consistent with our gene expression data. In view of this report, it is not clear how the increased expression of VEGFR2 is responsible for the observed protective effect of gelsolin on endothelial cells. We observed a significant increase in the secretion of pro-inflammatory cytokines characteristic of SARS-CoV-2 infection and S-protein action [70]. These pro-inflammatory cytokines (IL-6, TNF-α, INF-γ) were reported to exert inhibitory effects on endothelial cell proliferation and migration in vitro [71–74]. Moreover, IL-6 also reduces the action of VEGFa [75]. pGSN, when co-administered with S1, caused a decrease in the secretion of pro-inflammatory mediators, which might suggest that the pGSN, by its immunomodulatory effect, indirectly limits the S1-mediated inhibition of endothelial cell migration.

Induction and modulation of downstream SARS-CoV-2 signaling in the presence of pGSN creates the primary modulation of the innate and acquired immune response, which in turn reduces the pro-inflammatory response induced by S1. Among them are phosphoinositide 3-kinase (PI3K), mitogen-activated protein kinase (MAPK), protein kinase B (Akt), and nuclear factor-kappa B (NF-κB), and mammalian target of rapamycin (mTOR) [59, 60, 76–78]. In our study, pGSN, when simultaneously administered with SARS-CoV-2 S1 protein, caused significant inhibition of the PI3K/AKT downstream signaling, possibly through direct binding with PIP2, a phospholipid crucial for AKT activation. Plasma gelsolin is recognized as a biomarker of inflammation that can inhibit inflammatory responses by directly binding to products of bacterial origin, e.g., lipopolysaccharide and lipoteichoic acid [79–81]. To date, our current finding is the only report demonstrating an anti-inflammatory effect of plasma gelsolin, unrelated to its ability to bind pro-inflammatory mediators before their stimulation of a signaling pathways such as these mediated by activation of TLRs.

## Conclusions

pGSN significantly reduced the permeability of vessels forming the blood–brain barrier in 2D and 3D models. By impeding NF-κB-dependent signaling pathways, pGSN decreased the secretion of early pro-inflammatory cytokines (IL-6, L-8, TNF-α). In addition, pGSN promoted hCMEC/D3 cell migration in a wound-healing assay and inhibited the S1-induced breakdown of tight junction-forming proteins (ZO-1, Occludin, Claudin-5). Therefore, we propose that the administration of recombinant plasma gelsolin could serve as a promising tool to develop a new therapeutic strategy against SARS-CoV-2-mediated inflammation, especially those associated with BBB disruption. In conclusion, our results demonstrate that pGSN protects the blood–brain barrier changes occurring in response to SARS-CoV-2 Spike protein S1. pGSN may provide novel insights and strategies for the therapy of patients suffering from SARS-CoV-2 infection, especially in subjects with neurological manifestations.

## Supplementary Information

**Additional file 1: Table S1. **List of primary antibodies used for immunofluorescence studies and Western blotting experiments. **Figure S1.** Thrombin [5U] and human albumin [10 nM] was used as a positive and negative control, respectively, for Dextran-FITC permeability of the BBB. The fluorescence intensity of Dextran-FITC was measured in the lower chamber. The data represent the mean ± SEM of four independent experiments (N=4, two inserts per condition each time). * and ^ indicate statistical significance at p ≤ 0.05 compared to CT and S1, respectively by one-way ANOVA and Tukey post hoc test. **Figure S2. **Western blot bands (Panel A) and VE-cadherin and β-catenin expression fold change (Panel B). The data represent the mean ± SEM of four independent experiments (N=4). * and ^ indicate statistical significance at p ≤ 0.05 compared to CT and S1, respectively by one-way ANOVA and Tukey post hoc test. **Figure S3. **Log_2_FC of gene expression from Fig. 7A. Statistical significance at p ≤ 0.05 was assessed by one-way ANOVA and Tukey post hoc test. **Figure S4.** Raw images of Western blot.

## Data Availability

The datasets used and/or analyzed during this study are available from the corresponding authors on reasonable request.

## Abbreviations

pGSN: Plasma gelsolin
SARS-CoV-2: Severe acute respiratory syndrome coronavirus 2
BBB: Blood–brain barrier
COVID-19: Coronavirus disease
HCMEC: Human cerebral microvascular endothelial cells
TEER: Trans-endothelial electrical resistance
TJ and AJ: Tight and adhesive junctions
ACE2: Angiotensin converting enzyme 2
PDMS: Polydimethylsiloxane
