# Supplementary material for: Recombinant human plasma gelsolin reverses increased permeability of the blood–brain barrier induced by the spike protein of the SARS-CoV-2 virus
Source: J Neuroinflammation. 2022 Nov 24;19:282. doi: 10.1186/s12974-022-02642-4 (PMC9694610; doi:10.1186/s12974-022-02642-4)
Supplement: Supplementary file 1 — Additional file 1: Table S1. List of primary antibodies used for immunofluorescence studies and Western blotting experiments. Figure S1. Thrombin [5U] and human albumin [10 nM] was used as a positive and negative control, respectively, for Dextran-FITC permeability of the BBB. The fluorescence intensity of Dextran-FITC was measured in the lower chamber. The data represent the mean ± SEM of four independent experiments (N=4, two inserts per condition each time). * and ^ indicate statistical significance at p ≤ 0.05 compared to CT and S1, respectively by one-way ANOVA and Tukey post hoc test. Figure S2. Western blot bands (Panel A) and VE-cadherin and β-catenin expression fold change (Panel B). The data represent the mean ± SEM of four independent experiments (N=4). * and ^ indicate statistical significance at p ≤ 0.05 compared to CT and S1, respectively by one-way ANOVA and Tukey post hoc test. Figure S3. Log2FC of gene expression from Fig. 7A. Statistical significance at p ≤ 0.05 was assessed by one-way ANOVA and Tukey post hoc test. Figure S4. Raw images of Western blot. [file 12974_2022_2642_MOESM1_ESM.pdf]

Table S1. List of primary antibodies used for immunofluorescence studies and Western blotting experiments.

Primary Antibodies	Supplier	Species	Type	Dilution	Reference
ZO-1	Invitrogen	Rabbit	Polyclonal	1:200	40-2300
Occludin	Invitrogen	Rabbit	Polyclonal	1:200	40-4700
Claudin 5	Invitrogen	Mouse	Monoclonal	1:500	35-2500
VE-cadherin	Invitrogen	Mouse	Monoclonal	1:500	14-1449-82
β-catenin	Invitrogen	Mouse	Monoclonal	1:300	13-8400
β-actin	Sigma Aldrich	Mouse	Monoclonal	1:5000	A5441
VEGFR2	Invitrogen	Rabbit	Monoclonal	1:500	MA5-15157

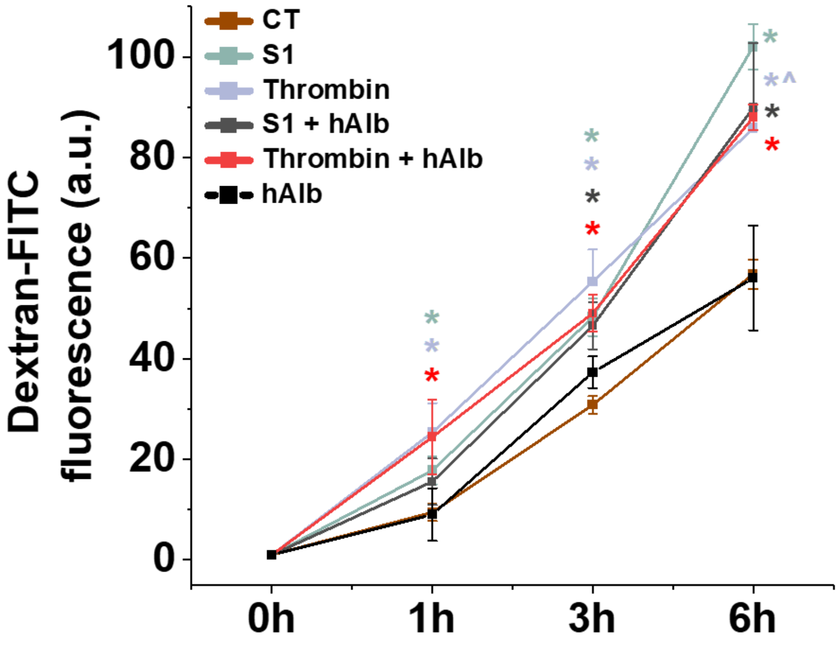

Figure S1. Thrombin [5U] and human albumin [10 nM] was used as a positive and negative control, respectively, for Dextran-FITC permeability of the BBB. The fluorescence intensity of Dextran-FITC was measured in the lower chamber. The data represent the mean ± SEM of four independent experiments (N=4, two inserts per condition each time). * and ^ indicate statistical significance at $p \leq 0.05$ compared to CT and S1, respectively by one-way ANOVA and Tukey post hoc test.

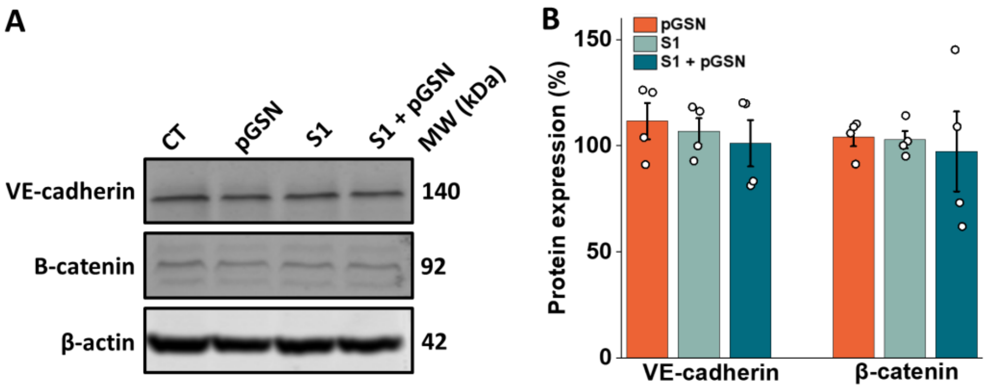

Figure S2. Western blot bands (Panel A) and VE-cadherin and β-catenin expression fold change (Panel B). The data represent the mean ± SEM of four independent experiments (N=4). * and ^ indicate statistical significance at $p \leq 0.05$ compared to CT and S1, respectively by one-way ANOVA and Tukey post hoc test.

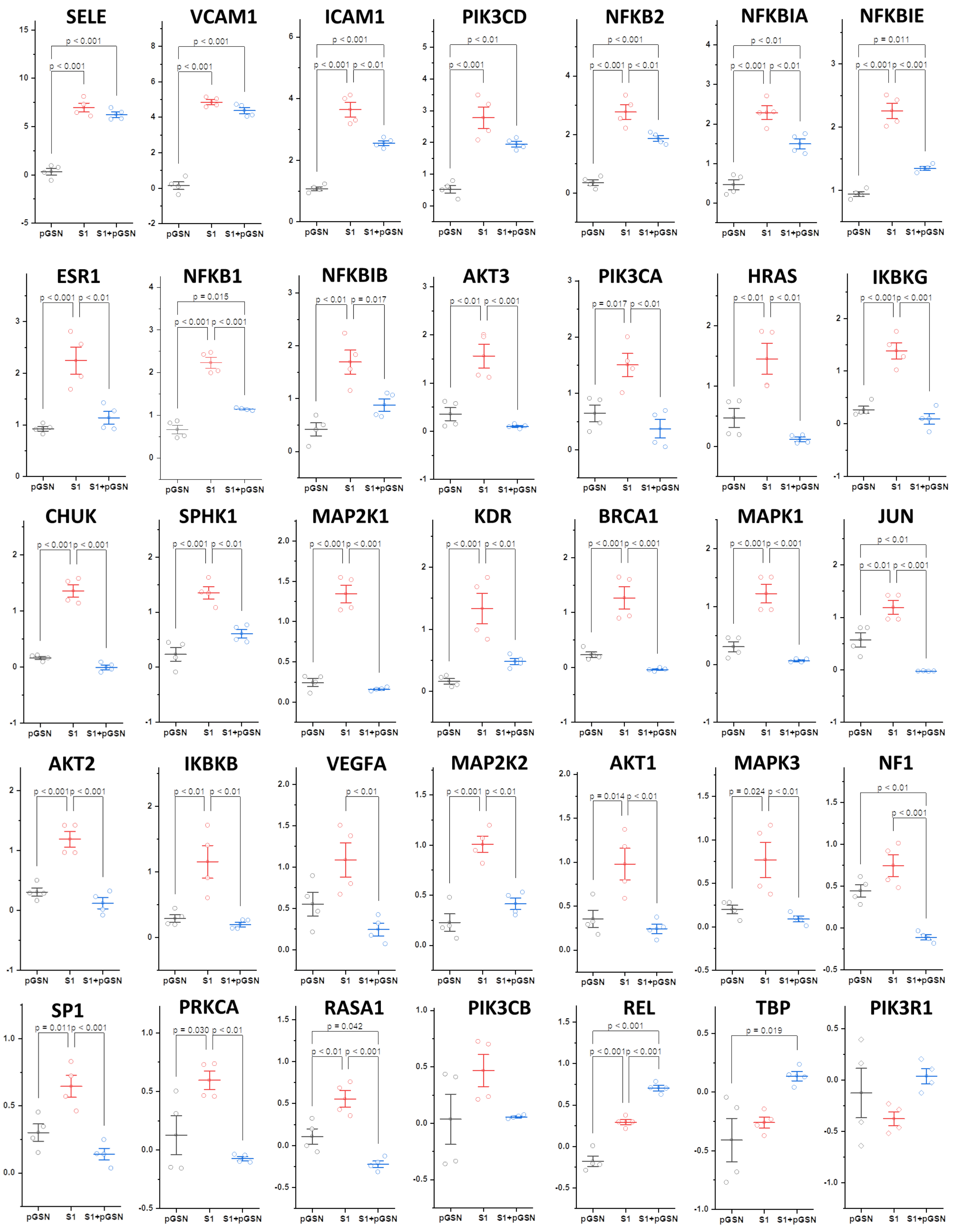

Figure S3. Log_2FC of gene expression from Fig. 7A. Statistical significance at $p \leq 0.05$ was assessed by one-way ANOVA and Tukey post hoc test.

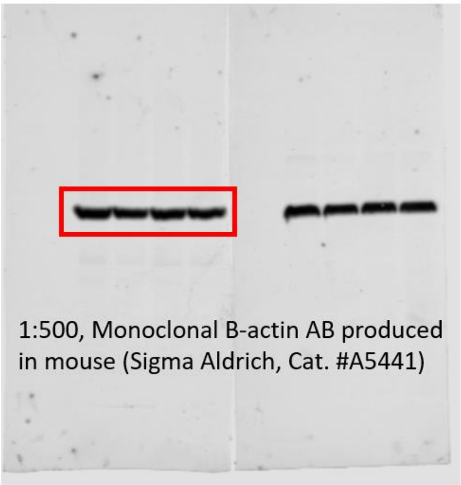

Full unedited blot of B-actin used in Figure 6A, and Supplemental Figure 2.

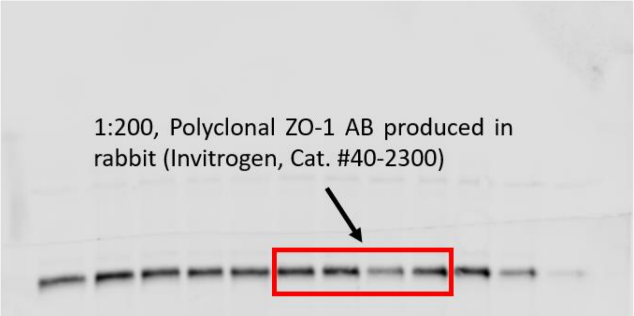

Full unedited blot of ZO-1 used in Figure 6A.

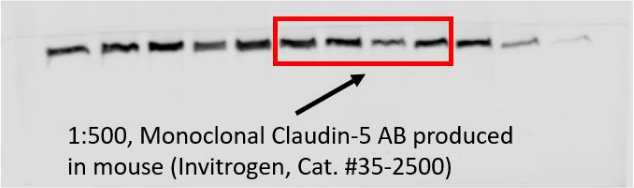

Full unedited blot of Claudin 5 used in Figure 6A.

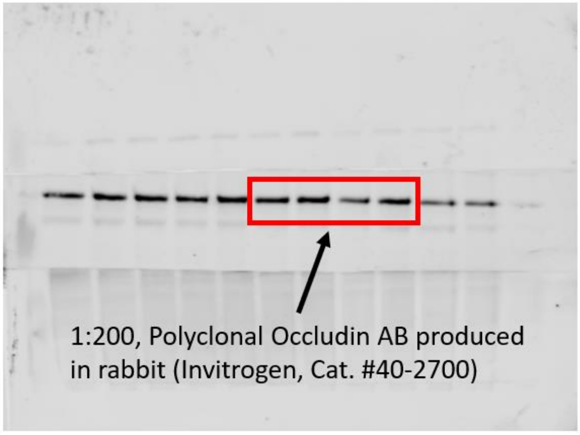

Full unedited blot of Occludin used in Figure 6A.

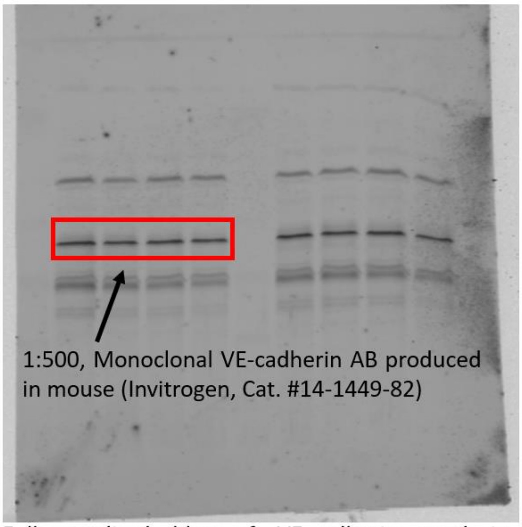

Full unedited blot of VE-cadherin used in Supplemental Figure 2.

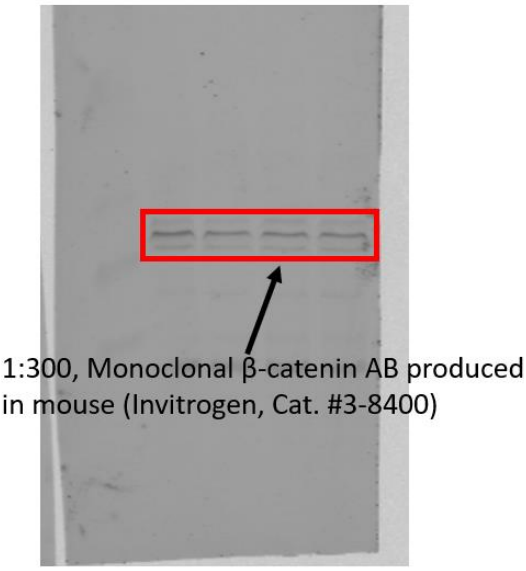

Full unedited blot of β-catenin used in Supplemental Figure 2.

Figure S4. Raw images of Western blot.
